# Simulation-based power calculations for planning a two-stage individual participant data meta-analysis

**DOI:** 10.1186/s12874-018-0492-z

**Published:** 2018-05-18

**Authors:** Joie Ensor, Danielle L. Burke, Kym I. E. Snell, Karla Hemming, Richard D. Riley

**Affiliations:** 1 Centre for Prognosis Research, Research Institute for Primary Care and Health Sciences, Keele University, Keele, Staffordshire ST5 5BG UK; 2 Institute of Applied Health Research, College of Medical and Dental Sciences, University of Birmingham, Edgbaston, Birmingham, B15 2TT UK

## Abstract

**Background:**

Researchers and funders should consider the statistical power of planned Individual Participant Data (IPD) meta-analysis projects, as they are often time-consuming and costly. We propose simulation-based power calculations utilising a two-stage framework, and illustrate the approach for a planned IPD meta-analysis of randomised trials with continuous outcomes where the aim is to identify treatment-covariate interactions.

**Methods:**

The simulation approach has four steps: (i) specify an underlying (data generating) statistical model for trials in the IPD meta-analysis; (ii) use readily available information (e.g. from publications) and prior knowledge (e.g. number of studies promising IPD) to specify model parameter values (e.g. control group mean, intervention effect, treatment-covariate interaction); (iii) simulate an IPD meta-analysis dataset of a particular size from the model, and apply a two-stage IPD meta-analysis to obtain the summary estimate of interest (e.g. interaction effect) and its associated *p*-value; (iv) repeat the previous step (e.g. thousands of times), then estimate the power to detect a genuine effect by the proportion of summary estimates with a significant *p*-value.

**Results:**

In a planned IPD meta-analysis of lifestyle interventions to reduce weight gain in pregnancy, 14 trials (1183 patients) promised their IPD to examine a treatment-BMI interaction (i.e. whether baseline BMI modifies intervention effect on weight gain). Using our simulation-based approach, a two-stage IPD meta-analysis has < 60% power to detect a reduction of 1 kg weight gain for a 10-unit increase in BMI. Additional IPD from ten other published trials (containing 1761 patients) would improve power to over 80%, but only if a fixed-effect meta-analysis was appropriate. Pre-specified adjustment for prognostic factors would increase power further. Incorrect dichotomisation of BMI would reduce power by over 20%, similar to immediately throwing away IPD from ten trials.

**Conclusions:**

Simulation-based power calculations could inform the planning and funding of IPD projects, and should be used routinely.

**Electronic supplementary material:**

The online version of this article (10.1186/s12874-018-0492-z) contains supplementary material, which is available to authorized users.

## Background

Individual patient data (IPD) meta-analysis involves obtaining and then synthesising the raw, individual level-data from multiple studies. The approach has become increasingly common over the past decade [1–3], due to the increasing willingness (and expectation [4]) of collaborators to share their IPD in order to answer questions previously unconsidered or not powered in their primary studies. One typical question is whether a patient-level characteristic modifies a treatment effect, in order to identify subgroups of patients who may be at greater benefit (or harm) than others. Such stratified medicine is a major interest of clinical decision makers and pharmaceutical companies, looking to identify those populations in whom treatment is more effective (or less harmful) [5]. A single trial is usually underpowered for this purpose. Brookes et al. [6] show that if a single trial has 80% power to detect a particular treatment effect (across all patients), then its power to detect an interaction (with a binary covariate) with the same magnitude as the overall treatment effect will only be 29%. To ensure 80% power to detect the interaction, the sample size in a single trial needs to be increased by approximately four times. Furthermore, to have 80% power to detect an interaction term half the size of the overall treatment effect there needs to be an approximately 16-fold increase in sample size. Therefore a project that pools the IPD from multiple trials is highly appealing to funders to substantially increase the power to detect genuine treatment-covariate interactions.

However, IPD meta-analyses are both time-consuming and expensive to perform, requiring significant resources to obtain, clean and harmonise the IPD from relevant trials before then synthesising them; a process that can take months or even years [7, 8]. Therefore, before embarking on an IPD project, researchers and funders should ensure that it is likely to be worth the effort. In particular, how many studies are likely to provide their IPD and, based on this, what is the potential power of the planned IPD meta-analysis? In our experience, power calculations and sample size justifications are rarely reported in IPD meta-analysis protocols or publications. Researchers are perhaps grateful for whatever IPD can be obtained, and appeal to any IPD meta-analysis adding value over a single trial. However, if it was known in advance that IPD from a particular number of studies would only increase power to 50%, then researchers and funders may think twice before undertaking the IPD project. Conversely, if a potential IPD meta-analysis increases the power to over 80%, then funders will be more reassured that the IPD project is worth resourcing. Power calculations could also reveal which studies contribute most to the power, and thus direct how much IPD is needed and from which studies, although this last point is potentially contentious.

Formal power calculations for an IPD meta-analysis are non-trivial and depend on many factors, which perhaps explains why they are currently neglected. The IPD cannot be considered as coming from a single trial, and thus sample size calculations must account for the clustering of patients within trials and the potential heterogeneity (e.g. in baseline risk and treatment effects) between-trials. Also, the power depends on the choice and specification of analysis model (e.g. covariates to be included, number of parameters, magnitude of effects), and the parameter estimation method, amongst other factors. Therefore, simple algebraic solutions are not straightforward unless simplifying conditions are made [9–11]. For this reason, Kontopantelis et al. previously proposed a simulation-based approach, where IPD meta-analysis datasets are simulated multiple times based on a chosen data-generating mechanism (including numbers of studies, effect sizes, and heterogeneity), and then a chosen one-stage IPD meta-analysis model is applied to each dataset, with subsequent results (e.g. estimates and confidence intervals) summarised over the multiple analyses [12]. In particular, the proportion of all simulations that give a *p*-value < 0.05 can be calculated, to give an estimate of the power.

Complementary to this work, in this paper we also propose simulation-based power calculations but within a two-stage IPD meta-analysis framework, rather than a one-stage. The two-stage approach is more common in practice, as the second stage enables meta-analysis models (such as inverse variance weights) and estimation methods (such as DerSimonian and Laird [13] or restricted maximum likelihood, REML) that are familiar to those working in the meta-analysis field. Also, it avoids convergence problems that are often more problematic for one-stage models (due to the inclusion of many study stratification terms and/or multiple random effects), and enables novel approaches (such as Hartung-Knapp Sidik-Jonkmann, HKSJ [14, 15]) to deriving confidence intervals that account for uncertainty in variance estimates. Crucially, it also automatically avoids ecological bias, which occurs in one-stage models when a treatment-covariate interaction is included without separating out individual-level associations from across-study associations [16, 17].

Below, we describe our new proposal and apply it to a real IPD meta-analysis of randomised trials in pregnancy, where the aim is to examine an interaction between baseline BMI and treatment effect. This illustrates how to tailor power calculations to the IPD meta-analysis at hand, using prior information (e.g. from published articles) and context-specific knowledge. The article is structured as follows. Section 2 briefly explains the two-stage approach to an IPD meta-analysis of continuous outcomes from randomised trials. Section 3 then outlines our simulation-based approach to power calculations, and Section 4 then details its application to the pregnancy example. Section 5 concludes with discussion, including how to extend to continuous and time-to-event outcomes.

## Methods

### The two-stage approach to IPD meta-analysis

We now introduce the two-stage approach to IPD meta-analysis of continuous outcomes, which was recently described by Burke et al. [18]

#### First stage

Let us assume that there are *i* = 1 to *K* randomised trials for the IPD meta-analysis and that a treatment effect is of interest. In the two-stage approach, usually the first stage involves a separate analysis in each study to derive the *K* treatment effect estimates and their variances, using an appropriate method chosen by the meta-analyst. In particular, a suitable regression model can be used for the outcome of interest, as now described.

If the outcome is continuous (weight, say) then one may use, for example, maximum likelihood (ML) estimation to fit an appropriate linear regression in each study separately. The ideal approach is an analysis of covariance (ANCOVA) model [19], which regresses the final value at end of follow-up, *y*_*Fij*_, and adjusts for baseline value, *y*_*Bij*_, and treatment (*x*_*ij*_ = 0/1 for participants in the control/treatment group) for the *j*^th^ participant in the *i*^th^ trial, as follows:

$$ {y}_{Fij}={\alpha}_i+{\delta}_i{y}_{Bij}+{\theta}_i{x}_{ij}+{e}_{ij} $$

1 $$ {e}_{ij}\sim N\left(0,{\sigma}_i^2\right) $$

In this model, α_*i*_ is the intercept (the expected final value in the control group for those with a baseline value of zero), *δ*_*i*_ is the expected effect on the final value for a 1-unit increase in the baseline value, *θ*_*i*_ is the treatment effect (the mean difference in weight between treatment groups after adjusting for baseline value), and *σ*_*i*_^*2*^ is the residual variance of the responses after accounting for the treatment effect and baseline value. As this model is fitted to each study separately, the true values of all parameters are naturally allowed to be different in each study (hence the *i* subscripts).

Although ANCOVA is preferred, sometimes baseline values are not provided in available IPD studies, and therefore alternative analyses are required, such as a final score model or a change score model. A final score model is the same as model (1), except without the *δ*_*i*_*y*_*Bij*_ term. The change score model is sensible when only the change score for each patient (${\mathit{\mathsf{y}}}_{\mathit{\mathsf{ij}}}$, say) is provided in the IPD, such as the weight gain during pregnancy from baseline (e.g. first consultation during pregnancy) to end of follow-up (e.g. last consultation before birth). The change score is then regressed against the treatment effect:

$$ {y}_{ij}={\alpha}_i+{\theta}_i{x}_{ij}+{e}_{ij} $$

2 $$ {e}_{ij}\sim N\left(0,{\sigma}_i^2\right) $$

In this model, α_*i*_ is the intercept (e.g. the expected weight gain in the control group), *θ*_*i*_ is the treatment effect (the mean difference in weight gain between treatment groups), and *σ*_*i*_^*2*^ is the residual variance of the responses after accounting for the treatment effect. It is worth noting that where interest lies in the change rather than final score, the change score model can also be adjusted for baseline to accurately estimate the treatment effect and its uncertainty.

Further baseline covariates might also be included in eqs. (1) and (2) in order to increase power or to adjust for baseline confounding. Indeed, an IPD meta-analysis project is usually initiated in order to go beyond the overall treatment effect, and examine how baseline covariates are associated with (interact with) treatment effect, in order to identify effect modifiers. For example, to examine the interaction between baseline BMI measured as a continuous variable and treatment effect (i.e. a treatment-BMI interaction), eq. (1) can be modified to,

$$ {y}_{Fij}={\alpha}_i+{\delta}_i{y}_{Bij}+{\beta}_i{BMI}_{ij}+{\theta}_i{x}_{ij}+{\lambda}_i\left({x}_{ij}\times {BMI}_{ij}\right)+{e}_{ij} $$

3 $$ {e}_{ij}\sim N\left(0,{\sigma}_i^2\right) $$

and eq. (2) modified to

$$ {y}_{ij}={\alpha}_i+{\beta}_i{BMI}_{ij}+{\theta}_i{x}_{ij}+{\lambda}_i\left({x}_{ij}\times {BMI}_{ij}\right)+{e}_{ij} $$

4 $$ {e}_{ij}\sim N\left(0,{\sigma}_i^2\right) $$

where the interaction term, *λ*_*i*_, denotes the mean increase in treatment effect for a 1-unit increase in the baseline BMI value. Estimation of eqs. (3) or (4) in each trial then provides the meta-analyst with *K* treatment-covariate interaction estimates (and their variances) ready for the second stage. Although continuous variables such as BMI, and interactions with BMI, could alternatively be modelled as categorical or with non-linear trends, in this article we generally assume that a linear relationship is appropriate. However, our approach can easily be adapted to situations where non-linear trends are considered more plausible.

#### Second stage

Following estimation of an equation such as (1) to (4) in each trial separately, the meta-analyst obtains *K* parameter estimates of interest. For example, eqs. (1) to (2) would provide treatment effect estimates,${\widehat{\theta}}_i$, and their variances, Var(${\widehat{\theta}}_i$); whilst eqs. (3) and (4) would provide interaction effect estimates, ${\widehat{\lambda}}_i$, and their variances, Var(${\widehat{\lambda}}_i$). These can now be combined in the second stage of the IPD meta-analysis. Let us focus on pooling treatment-covariate interactions (${\widehat{\lambda}}_i$), as these are usually the primary focus for an IPD meta-analysis of randomised trials. However, what follows could equally apply to any parameter estimate of interest, such as a treatment effect or a prognostic factor effect.

A meta-analysis model is chosen to pool the interaction estimates, ${\widehat{\lambda}}_i$, typically assuming that the true interaction is either fixed (common) or random across studies. The fixed effect model assumes that ${\widehat{\lambda}}_i$are all estimates of the same underlying interaction effect in all studies, represented as *λ*. It can be written generally as [20],

5 $$ {\widehat{\lambda}}_i\sim N\left(\lambda, Var\left({\widehat{\lambda}}_i\right)\right) $$

where the *Var*(${\widehat{\lambda}}_i$) estimates are also taken from the first stage, and usually assumed known. The most common method to estimate *λ* is the inverse variance method, which provides a weighted average, where the weight of each trial, *w*_*i*_, is defined as [21],

6 $$ {w}_i=\frac{1}{\mathit{\operatorname{var}}\left({\widehat{\lambda}}_i\right)} $$

and the pooled interaction effect, *λ*, and its variance are calculated by:

7 $$ \widehat{\lambda}=\frac{\sum_{i=1}^K{\widehat{\lambda}}_i{w}_i}{\sum_{i=1}^K{w}_i} $$

8 $$ \mathit{\operatorname{var}}\left(\widehat{\lambda}\right)=\frac{1}{\sum_{i=1}^K{w}_i} $$

The random effects model allows for between-study variation, *τ*^*2*^, in the true interaction effect, and makes the assumption that the different studies are estimating different, yet related, interaction effects. The random effects model can be written generally as [20],

$$ {\widehat{\lambda}}_i\sim N\left({\lambda}_i, Var\left({\widehat{\lambda}}_i\right)\right) $$

9 $$ {\lambda}_i\sim N\left(\lambda, {\tau}^2\right) $$

where the *Var*(${\widehat{\lambda}}_i$) estimates are again typically assumed known, and the true interaction effect in the *i*^th^ trial, *λ*_*i*_, is assumed normally distributed about an average interaction effect, *λ*, with between-study variance, *τ*^*2*^. Equation (9) reduces to equation (5) when *τ*^*2*^ equals zero. To obtain meta-analysis results, an inverse variance approach can again be taken but with the weights of each trial now adjusted to incorporate an estimate of *τ*^*2*^:

10 $$ {w}_i^{\ast }=\frac{1}{\mathit{\operatorname{var}}\left({\widehat{\lambda}}_i\right)+{\widehat{\tau}}^2} $$

Then, the estimate of the summary interaction effect and its variance are calculated using:

11 $$ \widehat{\lambda}=\frac{\sum_{i=1}^K{\widehat{\lambda}}_i{w}_i^{\ast }}{\sum_{i=1}^K{w}_i^{\ast }} $$

12 $$ \mathit{\operatorname{var}}\left(\widehat{\lambda}\right)=\frac{1}{\sum_{i=1}^K{w}_i^{\ast }} $$

There is ongoing debate about the best method to estimate *τ*^*2*^ [15, 22]. Traditionally, the most common method of estimating *τ*^*2*^ is the non-iterative, non-parametric methods of moments (MoM) estimator of DerSimonian and Laird [13]. However, other non-iterative estimators are available [23, 24], and iterative methods such as REML are also popular.

Following estimation of the chosen meta-analysis model, a standard 95% confidence interval for *λ* can be calculated as $\widehat{\lambda}$ ± 1.96$\sqrt{\mathit{\operatorname{var}}\left(\widehat{\lambda}\right)\ }.$However, this has been criticised because it ignores uncertainty in variance estimates, in particular ${\widehat{\tau}}^2$, and thus leads to inappropriate coverage of confidence intervals (inflated type I errors) [15, 25]. To address this, alternative methods have been proposed for deriving 95% confidence intervals for the summary effect; in particular, the HKSJ approach provides a modification to the variance (*var*_*HKSJ*_) of the summary estimate [14, 26–29], and derives 95% confidence intervals by $\widehat{\lambda}\pm \left({t}_{0.975,k-1}\sqrt{{\mathit{\operatorname{var}}}_{HKSJ}\left(\widehat{\lambda}\right)}\right)$, which are usually appropriately wider than the standard approach.

### Simulation-based power calculations for a two-stage IPD meta-analysis of continuous outcomes

We now propose our simulation-based approach to power calculations, which utilise the two-stage IPD meta-analysis framework. The general premise is that an IPD meta-analysis dataset is simulated and then a two-stage meta-analysis performed. This is repeated many (e.g. thousands of) times (*m*, say), and each time the resulting summary estimates, confidence intervals and *p*-values are stored. Based on a traditional frequentist paradigm, power can then be estimated by calculating the proportion of times the summary estimate was statistically significant (e.g. as defined by the associated 95% confidence interval excluding the null value, or equivalently an associated *p*-value < 0.05). The general step-by-step process is now outlined.

#### Step (i): Specification of a statistical model in each trial

Firstly, a data generating model needs to be assumed for each trial. Ideally, this should be in accordance with the model that will be fitted in the first stage of the two-stage IPD meta-analysis. For example, ANCOVA model (1) might be assumed when interest lies in a continuous outcome and a treatment effect, or model (3) if the focus is a treatment-covariate interaction effect. However, if baseline values are potentially not available, change score models (2) and (4) may be alternatively assumed. The choice may also be influenced by the reported information in the publications, for example in regard whether final score or change score summary statistics are given, as these inform step (ii) below. Also, it may help to centre covariates about their trial-specific mean value, to ease the interpretation of the parameters for step (ii).

#### Step (ii): Choose parameter values for the statistical model and study characteristics (e.g. number of patients, covariate distributions)

Next, sensible parameter values need to be specified for the chosen model. Table 1 provides a summary of the input values required for continuous outcomes, respectively, when adopting models (1) to (4) as the statistical model within each trial. This includes specifying the magnitude of trial intercepts (control group responses / baseline risk), the magnitude and distribution of treatment and interaction effects, and the magnitude of residual and between-study variances. Also required are characteristics of the trials themselves. That is, the number of trials promising IPD, the number of patients therein, and the distribution of covariate values (e.g. proportion in the treatment and control groups; mean and standard deviation of baseline BMI in each trial; etc).

**Table 1:** Example of inputs required for simulation-based power calculations for an IPD meta-analysis of randomised trials with a continuous outcome

When considering the power of a summary (overall) treatment effect with model (1) or (2) used as the data generating model in the first stage:	
• Number of simulations to conduct (recommend at least 1000)	
• Number of trials in the IPD meta-analysis	
• Number of patients in each trial, and proportion treated	
• Method for estimating the treatment effect in each study separately	
• Magnitude of control group mean outcome in each trial (‘baseline risk’)	
• Between-trial distribution and magnitude of treatment effects, e.g. normal with a particular mean (summary) effect and between-trial variance (plus any between-trial correlation of baseline risks and treatment effects, if considered relevant)	
• Magnitude of residual variance in each trial	
• For ANCOVA model: distribution and magnitude of baseline continuous values in each trial e.g. normal with particular mean and variance	
• For ANCOVA model: between-trial distribution and magnitude of the prognostic effect of the baseline continuous values, e.g. normal with particular mean and variance	
• Approach to use in second stage of the two-stage IPD meta-analysis to pool effect estimates: e.g. fixed effect model or random effects model	
• Approach to derive confidence intervals and *p*-values (e.g. standard normal-based method, Hartung-Knapp Sidik-Jonkman, etc)	
Additionally, when considering the power of a treatment-covariate interaction with models (3) or (4) used as the data generating model in the first stage:	
• Analysis model and method for estimating the interaction effect in each study separately	
• Distribution and magnitude of covariate values in each trial; e.g. normal with chosen mean and variance for a continuous covariate, or Bernoulli for a binary covariate with a chosen probability of being a 1.	
• Between-trial distribution and magnitude of treatment-covariate interaction effect, e.g. normal with a particular (summary) mean effect and between-trial variance	

Though this may sound onerous, it is usual to know which trials may provide (or could be approached for) their IPD. Then, aggregate information (summary statistics) available in trial publications and reports can be used to inform the values of parameters and characteristics within trials. This is illustrated in detail in the worked example in Section 4.

#### Step (iii): Generate an IPD meta-analysis dataset and undertake a two-stage IPD meta-analysis

Following steps (i) and (ii), an IPD meta-analysis dataset of a given number of trials and patients can be generated based on the statistical model and characteristics specified, using the simulation approach. This requires user-written software to randomly generate the IPD meta-analysis dataset based on the conditions given. Our supplementary material provides Stata code to illustrate how this can be done for the pregnancy example presented in Section 4 (see Additional file 1).

Once the IPD meta-analysis dataset is generated, a two-stage IPD meta-analysis is then applied as outlined in the previous section, to obtain the summary effect estimate of interest, and its associated confidence interval and *p*-value. The exact approach depends on the preference of the user. For example, after model (4) is applied to each trial separately, the second stage could implement either model (5) or (9) to pool the trial interaction estimates using either a fixed effect or random effects analysis, respectively. Confidence intervals and *p*-values of summary estimates can then be calculated, for example using the standard normal-based approach or the HKSJ method.

#### Step (iv): Repeat multiple times and evaluate power

Step (iii) is then repeated many (thousands of) times, until *m* summary effect estimates, confidence intervals and *p*-values are obtained. Assuming the IPD were simulated according to a genuine effect (e.g. a non-zero mean difference between treatment and control, or a non-zero treatment-covariate interaction), the proportion of these *m* results that were statistically significant gives an estimate of the power of the IPD meta-analysis. Thus, it reveals the probability that, if the IPD meta-analysis project could be repeated identically many times, the summary result would detect (with statistical significance) the genuine effect. The definition of statistical significance is of course arbitrary. Usually *p* < 0.05 (or equivalently the 95% confidence excluding the null value) will be used, but the user can adapt this if desired (e.g. *p* < 0.01), for example for multiple testing. Once the power estimate is obtained, a 95% confidence interval for the power can also be calculated (for example using an exact method [30]), which will become narrower as *m* increases.

It is also sensible for steps (i) to (iv) to be repeated after adopting different (yet still realistic) parameter values, to ascertain if and how power changes accordingly. For example, initially the assumed model may assume no between-trial heterogeneity on treatment or interaction effects, but this may be relaxed in subsequent simulations. This will be illustrated in Section 4.

### Applied example: Power of a planned IPD meta-analysis of trials of interventions to reduce weight gain in pregnant women

We now illustrate the key concepts through an applied example. In this example, our aim is to reflect the process researchers go through when considering or planning an IPD meta-analysis project. We assume that a clinical question has been identified and an IPD meta-analysis project is desired to address it. Additionally, a set of trials has been identified (and potentially promised their IPD) and aggregate data (summary statistics) for these trials have been published. The researchers want to know, *in advance of collecting IPD*, whether an IPD meta-analysis of these trials is likely to be powered to answer the clinical question at hand.

#### Background for applied example

Thangaratinam et al. [31] performed a systematic review to investigate the effects of weight management interventions on maternal and fetal outcomes. One of the primary outcomes was maternal weight gain and their aggregate data meta-analysis of 30 randomised trials showed a significant average reduction in weight gain of 0.97 kg (95% CI: 0.34 kg to 1.60 kg reduction) for lifestyle interventions compared with control. However, there was a large amount of between-study heterogeneity, with an I-squared statistic of 87% and ${\widehat{\tau}}^2$of 1.87. Therefore, a major recommendation of Thangaratinam et al. was that an “IPD meta-analysis is needed to provide robust evidence on the differential effect of intervention in various groups based on BMI, age, parity, socioeconomic status and medical conditions in pregnancy”. That is, IPD was needed to examine potential treatment-covariate interactions.

In response to this, in 2012 the Weight Management in Pregnancy International IPD Collaboration (i-WIP) was established to share IPD from multiple randomised trials, and the National Institute for Health Research (NIHR) Health Technology Assessment (HTA) programme subsequently funded the project. At the time of developing the grant application, 14 of the trials (containing 1183 patients) included in the aforementioned aggregate data meta-analysis had provisionally agreed to provide their IPD. These are summarised in Table 2, including information about the weight gain in each treatment group, and the distribution of baseline BMI values. No formal power calculation was originally performed for the i-WIP grant application, but it was noted that in order to detect treatment-covariate interactions “our IPD meta-analysis provides an efficient way to substantially increase the sample size, without the need for a new trial”.

**Table 2:** Summary information, available prior to the IPD meta-analysis, about 14 trials that were included in the aggregate data meta-analysis of Thangaratinam et al. [31] and had promised their IPD at the time of the IPD meta-analysis grant application

		Intervention group					Control group						
Author	Year	n	Mean weight gain (kg)	SD of weight gain	Mean BMI at baseline	SD of BMI	n	Mean weight gain (kg)	SD of weight gain	Mean BMI at baseline	SD of BMI	Intervention effect (difference in weight gain)	95% CI
Wolff	2008	23	6.60	5.50	34.90	4.00	27	13.30	7.50	34.60	3.00	−6.70	(− 10.31, −3.09)
Landon	2009	476	2.80	4.50	30.10	5.00	455	5.00	3.30	30.20	5.10	−2.20	(−2.71, −1.69)
Rae	2000	67	11.56	10.80	37.90	0.70	58	9.68	11.04	38.00	0.70	1.88	(−1.96, 5.72)
Guelinck	2010	42	9.80	7.60	33.75	3.79	43	10.60	6.90	33.50	3.90	−0.80	(−3.89, 2.29)
Jeffries	2009	124	10.70	4.21	NA	NA	111	11.50	4.03	NA	NA	−0.80	(−1.85, 0.25)
Jackson	2010	163	15.15	5.50	NA	NA	164	15.24	6.67	NA	NA	−0.09	(−1.41, 1.23)
Hui	2006	24	14.20	5.30	23.40	3.90	21	14.20	6.30	25.70	6.30	0.00	(−3.43, 3.43)
Ong	2009	6	3.70	3.40	35.10	3.50	6	5.20	1.30	35.10	3.50	−1.50	(−4.41, 1.41)
Khaledan	2010	18	4.04	3.49	NA	NA	21	5.00	3.70	NA	NA	−0.96	(−3.22, 1.30)
Barakat	2009	72	11.50	3.70	24.30	0.50	70	12.40	3.40	23.40	0.50	−0.90	(−2.07, 0.27)
Haakstad	2009	52	13.00	4.00	NA	NA	53	13.80	3.80	NA	NA	−0.80	(−2.29, 0.69)
Hopkins	2010	47	8.20	3.49	25.50	4.30	37	8.00	3.70	25.40	2.90	0.20	(−1.35, 1.75)
Marquez-Sterling	2000	9	16.20	3.40	22.80	4.00	6	15.70	4.00	24.50	4.50	0.50	(−3.40, 4.40)
Yeo	2009	60	15.90	6.80	NA	NA	64	15.40	5.90	NA	NA	0.50	(−1.75, 2.75)

Retrospectively, we now consider how our simulation-based approach to power would have been useful to the i-WIP collaborators, to provide formal quantitative reassurance of the power of their planned IPD meta-analysis project. Here we focus on the power to detect a potential interaction between baseline BMI and intervention effect, which was one of the primary objectives of their study. The prior hypothesis was that those with high baseline BMI may benefit most from weight management interventions.

#### What is the power to detect a treatment-BMI interaction with 14 trials promising IPD?

We start by applying random effects meta-analysis model (9) to the 14 published intervention effect estimates shown in Table 2. This gives a summary mean difference of − 0.84 kg (95% CI: -1.63 to − 0.06), indicating an average reduction in weight gain of 0.84 kg by using an intervention rather than control. Heterogeneity was large, with an I-squared statistic of 63% and ${\widehat{\tau}}^2$of 1.1, with the latter estimated by the approach of DerSimonian and Laird (methods of moments) [13]. These results are very similar to those from the original aggregate data meta-analysis of 30 trials, suggesting the 14 trials are broadly representative of the original set of trials. Let us now apply our simulation-based approach, following the steps described in Section 3, to quantify the potential power to detect an interaction between baseline BMI and intervention effect using these 14 trials, with BMI measured as a continuous covariate and linear effects and interactions for BMI assumed correct.

### Methods for applied example

#### Step (i): Specification of the treatment-covariate model in each trial

The first step of the simulation approach is to define an underlying (data generating) model for each trial. It is preferable to keep this simple and reflect the analysis model that is likely to be used in the first stage of the IPD meta-analysis. As weight is a continuous outcome, we ideally wanted to consider an ANCOVA model (1). However, the summary statistics reported in each trial mainly focused on weight gain (rather than final weight), thus it was considered sensible to focus initially on eq. (4), to ease specification of parameter values in step (ii) (NB extension to ANCOVA is considered in Section 4.5). Thus the assumed model was as follows:

13 $$ {Y}_{ij}={\alpha}_i+{\beta}_i{\overline{\mathrm{BMI}}}_{ij}+{\theta}_i{x}_{ij}+{\lambda}_i\left({x}_{ij}\times {\overline{\mathrm{BMI}}}_{ij}\right)+{e}_{ij} $$

Here, *Y*_*ij*_ is the weight gain during pregnancy for patient *j* in trial *i*, and this is regressed against baseline BMI value (${\overline{\mathrm{BMI}}}_{ij}\Big)$, the treatment group (*x*_*ij*_), and the interaction between baseline BMI and treatment (${x}_{ij}\times {\overline{BMI}}_{ij}\Big)$. Note that ${\overline{BMI}}_{ij}$denotes the baseline BMI value for patient *j* centred about the mean baseline BMI in trial *i*. This specification greatly eases the interpretation and specification of model parameters in step (ii).

We also assumed that,

$$ {e}_{ij}\sim N\left(0,{\sigma}_i^2\right) $$

$$ {\beta}_i\sim N\left(\beta, {\tau}_{\beta}^2\right) $$

$$ {\theta}_i\sim N\left(\theta, {\tau}_{\theta}^2\right) $$

$$ {\lambda}_i\sim N\left(\lambda, {\tau}_{\lambda}^2\right) $$

such that the residuals (*e*_*ij*_) in each trial have a variance of ${\sigma}_i^2$, and the parameters of *β*_*i*_ (the effect of a 1-unit increase in baseline BMI on the mean control group weight gain), *θ*_*i*_ (the treatment effect for a patient with the mean baseline BMI) and *λ*_*i*_ (the effect of a 1-unit increase of baseline BMI above the mean baseline BMI on the treatment effect) are drawn from independent normal distributions with means (*β*, *θ*, *λ*) and variances (${\tau}_{\beta}^2$, ${\tau}_{\theta}^2$, ${\tau}_{\lambda}^2$). This is the simplest option, but of course different (and dependent) between-trial distributions could be assumed, but for parsimony the use of normal distributions was deemed sensible. If considered important, between-study correlation could also be included between the baseline risk (*α*_*i*_) and overall treatment effect (*θ*_*i*_).

#### Step (ii): Choose parameter values for the statistical model and study characteristics

In order to simulate IPD under this model structure, the next step was to specify the assumed magnitude of *α*_*i*_, *β*, *θ*, *λ*, ${\sigma}_i^2$, ${\tau}_{\alpha}^2$, ${\tau}_{\beta}^2$, ${\tau}_{\theta}^2$, and ${\tau}_{\lambda}^2$. Though this may seem onerous, it is relatively straightforward. A summary of our chosen parameter values is given in Table 3, and we now explain the justification.

**Table 3:** Parameter values and trial characteristics initially chosen for the simulation-based power calculations of the IPD meta-analysis of pregnancy trials

Parameter	Chosen values	Interpretation and justification
No. of trials	14	Number of studies included in a previous aggregate data meta-analysis that had promised their IPD
Sample sizes	50, 931, 125, 85, 235, 327, 45, 12, 39, 142, 105, 84, 15, 124	Total sample size: taken from original trial publications (could breakdown further into the number in control and treatment groups if unequal)
*α*_*i*_	13.30, 5.00, 9.68, 10.60, 11.50, 15.24, 14.20, 5.20, 5.00, 12.40, 13.80, 8.00, 15.70, 15.40	Mean weight gain in the control group: used values as stated in original trial publications
*β*	−0.28	Prognostic effect of BMI on weight gain: used estimate from a meta-regression of mean weight gain against mean baseline BMI in the control group
*θ*	−0.84	Mean treatment effect across trials: used summary estimate from random effects meta-analysis of published estimates from the 14 trials
*λ*	Various: −0.5, − 0.4, − 0.3, − 0.2, − 0.1, −.05, − 0.025, − 0.01	Magnitude of interaction: used range from extremely large to extremely small interaction effect
${\sigma}_i^2$	43.25, 15.57, 119.26, 52.69, 16.98, 37.37, 33.89, 6.63, 12.93, 12.63, 15.22, 12.93, 13.78, 40.53	Residual variance: used unweighted average of the variance values for treatment and control groups as stated in original trial publications
${\tau}_{\beta}^2$	0	Between-study variance of the prognostic effect of baseline BMI: set to zero for parsimony
${\tau}_{\theta}^2$	1.1	Between-study variance of overall treatment effect: used estimate from random effects meta-analysis of published estimates from the 14 trials
${\tau}_{\lambda}^2$	0	Between-study variance of interaction effect: set to zero for parsimony
Distribution of baseline BMI values	Study 1: N(34.75, 12.5) Study 2: N(30.15, 25.51) Study 3: N(37.95, 0.49) Study 4: N(33.63, 14.77) Study 7: N(24.55, 27.45) Study 8: N(35.1, 12.25) Study 10: N(23.85, 0.25) Study 12: N(25.45, 13.45) Study 13: N(23.65, 18.13) Other studies had a mean drawn from N(30, 2.5) and within-trial standard deviation set at 3.5	Distribution of key covariate of interest: assumed normality, with means and variances as stated in original trial publication, or if unavailable, values based on those observed from within and between other trials

Each *α*_*i*_ corresponds to the mean weight gain for control individuals with the mean BMI, which we considered similar to the mean weight gain in the control group, and was available for each trial (Table 2). For example, *α*_1_ was set to 13.3. The residual variance (${\sigma}_i^2$) in each trial was approximated from the standard deviation of weight gain values available from the publications (Table 2). For example, for trial 1, assuming that the residual variance would be the same for control and treatment groups, we took an average of 5.5^2^ and 7.5^2^, which is 43.25. We assumed that *θ* = − 0.84, which is the summary treatment effect estimate from the aforementioned meta-analysis of the 14 published estimates. Similarly, based on the estimated between-trial variability in treatment effects from this meta-analysis, we assumed that ${\tau}_{\theta}^2$ = 1.1.

It was considered sensible to have a parsimonious situation where ${\tau}_{\beta}^2$ and ${\tau}_{\lambda}^2$ were zero, such that there was no between-trial heterogeneity in the prognostic effect of baseline BMI or in the interaction effect (this latter assumption is relaxed in Section 4.4). A value for *β* was also needed. Using the nine trials with baseline BMI information, a random effects meta-regression of the mean weight gain versus the mean baseline BMI in the control group (weighted by the inverse of the variance of mean weight gain) was fitted, and this gave an association of − 0.28. We took this study-level association as a proxy for the individual-level association represented by *β*, which suggests that weight gain decreases by 0.28 kg for every unit increase in baseline BMI. This agrees with guidelines that recommend weight gain should be lower in those with a higher baseline BMI. In this way, the generation of an individual’s change in weight is now correlated with the baseline BMI (and thus baseline weight), as expected by definition. Furthermore, it allows individuals with a high BMI to be more likely to be amongst a small subset that actually lose weight during pregnancy, which is plausible given the reported magnitude of the standard deviations for weight gain relative to the mean value (Table 2).

Lastly, we needed to choose the magnitude of *λ*, our key parameter of interest in the IPD meta-analysis. Our specification of model (13) assumes that the interaction effect is linear, such that a 1-unit increase in baseline BMI modifies the treatment effect on weight gain by *λ*. Although categorical or non-linear relationships could alternatively be assumed [32], the linear effect was chosen for parsimony. The hypothesis that the treatment effect may be larger for those with a higher baseline BMI implies that *λ* would be negative. Rather than choosing a single value for *λ*, we repeated simulations for each of a range of values between − 0.01 and − 0.5, moving from small (and potentially not clinically important) to extremely large interaction effects. For example, if *λ* was -0.05, then for a ten unit increase in baseline BMI, there would be an extra 0.5 kg reduction in weight gain by using the intervention rather than the control.

The number of trials in the IPD meta-analysis was set at 14 trials, each containing the number of patients known (Table 2), with close to an even allocation of patients to treatment and control groups. The distribution of baseline BMI was also needed within each trial. For nine trials, the published data gave the mean baseline BMI and its standard deviation (Table 2), and we assumed a normal distribution for baseline BMI in these trials. For example, for trial 1, using the average observed values for the treatment and control groups, baseline BMI was assumed to be normally distributed with a mean of 34.75 and variance of 12.5. For the remaining five trials without BMI information, the mean baseline BMI was drawn from a normal distribution with a mean of 30 and standard deviation of 2.5, and a within-trial standard deviation of 3.5 was assumed; this was based on the range of baseline BMI values observed within and across the other nine trials.

#### Steps (iii): Generate an IPD meta-analysis dataset and undertake a two-stage IPD meta-analysis

We created a module within Stata that generated an IPD meta-analysis dataset containing 14 trials based on model (13) and the chosen set of parameter values and trial characteristics shown in Table 3. That is, in each trial, for each patient we randomly generated their treatment group (*x*_*ij*_), their baseline BMI value centred about the observed trial’s mean baseline BMI (${\overline{BMI}}_{ij}$), and their weight gain (*Y*_*ij*_).

This enabled us, within the same Stata module, to then immediately undertake a two-stage IPD meta-analysis. In the first stage, model (13) was fitted to each trial separately to produce the treatment-BMI interaction estimate and its variance; then, in the second stage a fixed effect meta-analysis model (model (5)) was used to pool the interaction estimates.

#### Step (iv): Repeat multiple times and evaluate power

Step (iii) was repeated until we had randomly generated 10,000 IPD meta-analysis datasets, each containing 14 trials. For each of the 10,000 datasets, the Stata module performed a two-stage IPD meta-analysis and the results were stored. This produced 10,000 summary treatment-BMI interaction estimates and their 95% confidence intervals and *p*-values (one for each IPD meta-analysis dataset). Confidence intervals were derived using the standard (normal-based) method. The power of the planned IPD meta-analysis was then calculated as the proportion of 10,000 meta-analyses where the summary interaction estimate was detected by a *p*-value < 0.05 (or equivalently a 95% confidence interval that did not contain the null value).

The Stata module to implement steps (i) to (iv) is provided in the supplementary material (see Additional file 1). This module allowed us to repeat steps (i) to (iv) for different assumed parameter values and model approaches. In particular, we also considered non-zero values of ${\tau}_{\lambda}^2$ and fitted a random effects meta-analysis model (9) in the second stage of the IPD meta-analysis, and rather calculated *p*-values and confidence intervals according to the HKSJ method, to examine if and how power was affected.

## Results

**Fig. 1:**
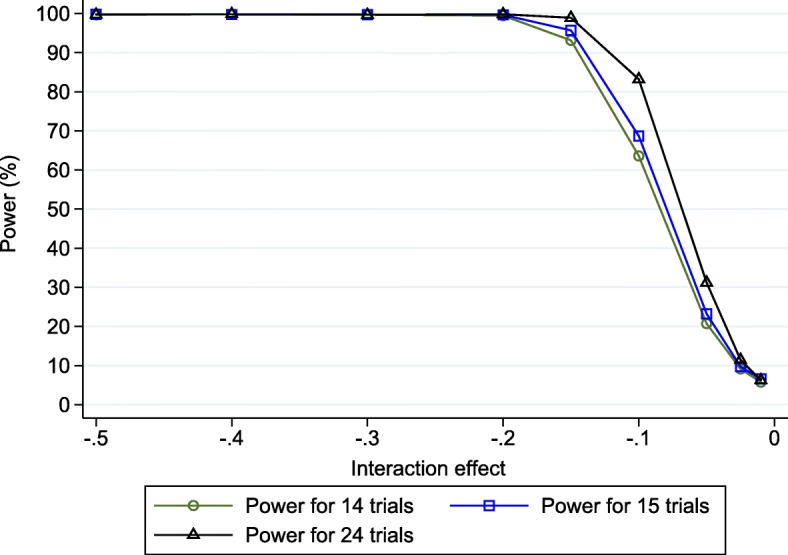
Simulation-based power estimates (based on 10,000 replications) for the planned IPD fixed effect meta-analysis* of either 14, 15 or 24 trials for detecting a treatment-BMI interaction effect (*λ*), across a range of values. * Based on using change score model (4) in each trial followed by fixed effect meta-analysis model (9)

Our simulation-based power estimates for the potential IPD meta-analysis are shown in Fig. 1, across the range of true interaction effects from − 0.01 to − 0.5. Power increases as the magnitude of the interaction estimate increases, which is to be expected as, other things being equal, a *p*-value becomes smaller as the estimate moves further from the null (which, here, is an interaction of zero).

Despite having IPD from 14 trials, including 2319 patients, the estimated power is less than 80% unless the true interaction effect is about − 0.15 or more. For example, for a true interaction effect of − 0.1, the power is estimated to be 63.6% (95% CI: 62.6%, 64.5%) because 6360 of the 10,000 simulated IPD meta-analyses produced a significant result. For a true interaction effect of − 0.05, the power reduces dramatically to just 20.7%. This indicates that the planned IPD meta-analysis may be underpowered to detect potentially clinically relevant treatment-BMI interactions.

Of note, the mean interaction estimates across each set of 10,000 simulations were almost identical to the true interaction effect, across the entire range from − 0.01 to − 0.5. Thus, the low power was not due to any systematic bias due to the IPD meta-analysis model or estimation process.

### Extension to consider obtaining IPD from additional trials

When faced with such findings of low power, researchers are then likely to enquire about whether additional IPD are available, and indeed how much IPD is required to adequately improve the power. In the i-WIP project, following discussion with collaborators, IPD were additionally promised from a further 10 trials that, for various reasons, were not included in the original published meta-analysis of aggregate data [31]. Given that the collection of IPD is potentially time-consuming and resource intensive [7, 8], a dilemma is whether IPD is needed from all of these 10 trials, or perhaps just a representative subset. Power calculations are helpful to resolve this. For illustration, here we consider two options: (i) adding IPD from just the largest of the 10 additional trials, which contained 399 patients; or (ii) adding IPD from all 10 additional trials (a total of 1761 additional patients). We repeated our simulation approach for each of these situations. Sample sizes for the 10 additional trials were known, but information was often lacking about other factors (e.g. the control group mean, or the distribution of baseline BMI) and so we sampled these from the distributions observed in others trials. For example, control group mean weight gain was sampled from ${\alpha}_i\sim N\left(\alpha, {\tau}_{\alpha}^2\right)$, with *α* and ${\tau}_{\alpha}^2$ set to 11 and 22 respectively, corresponding to their values from a random effects meta-analysis of the mean weight gain estimates for the control groups from the original 14 trials (Table 2).

The results are presented within Fig. 1, and show that adding IPD from further trials would increase the power as expected. However, adding just the IPD from the largest trial is not sufficient, as the power remains lower than typically desired at relevant values of the interaction effect. For example, with a true interaction effect of − 0.1 the IPD meta-analysis of 15 trials has an estimated power of 68.7% (67.8% to 69.6%), and with an interaction effect of − 0.05 it has an estimated power of only 23.2% (95% CI: 22.4% to 24.1%).

Findings based on adding IPD from all 10 additional trials are more promising. In particular, for a true interaction effect of − 0.1 the IPD meta-analysis of 24 trials has an estimated power of 83.2% (95% CI: 80.2% to 85.0%). This is above 80% for the first time, which is a threshold often used in power calculations for single randomised trials. Thus, there is large power to detect interaction effects of ≤ -0.1. However, the power to detect an interaction of size − 0.05 remains very low (31.2%). Therefore, if the true interaction effect is − 0.05, then the IPD meta-analysis is unlikely to have the power required even with 24 trials.

We note that sample size is not the only criteria that will impact upon a study’s contribution toward power. For a treatment-covariate interaction, the standard deviation of covariate values is also important [9]: other things being equal, those studies with larger variation in covariate values will have a greater contribution. For example, assuming a true interaction effect of − 0.1, if we remove the Barakat study from the IPD meta-analysis of 24 trials, the power estimate is lower than if we remove the Wolff study, even though the latter has far fewer patients. The reason is that the standard deviation of BMI values is substantially larger in the Wolff study (Table 2).

### Extension to random effects meta-analysis and alternative confidence interval derivations

The above power calculations assume a fixed effect meta-analysis of interaction estimates and no between-study heterogeneity on the interaction effect. Also, our confidence intervals and *p*-values for the intervention effect were derived using the standard normal-based approach, but options such as HKSJ are also possible, as previously mentioned [15].

We therefore repeated our power calculations for the IPD meta-analysis of 24 trials using the same parameter values as shown in Table 3, except with non-zero heterogeneity on the interaction effect (${\tau}_{\lambda}^2$ > 0) and with trial interaction estimates pooled using random effects meta-analysis model (9) via the DerSimonian and Laird approach. Confidence intervals and *p*-values were derived using the standard approach, but also using the HKSJ approach for comparison. We focus only on the situation where *λ* = − 0.1, as this was the critical value for an 80% power as identified from the fixed effect simulations. A range of values for *τ*_*λ*_was considered, from 0.01 to 0.05, which covered low heterogeneity to large heterogeneity relative to an interaction effect of − 0.1.

**Fig. 2:**
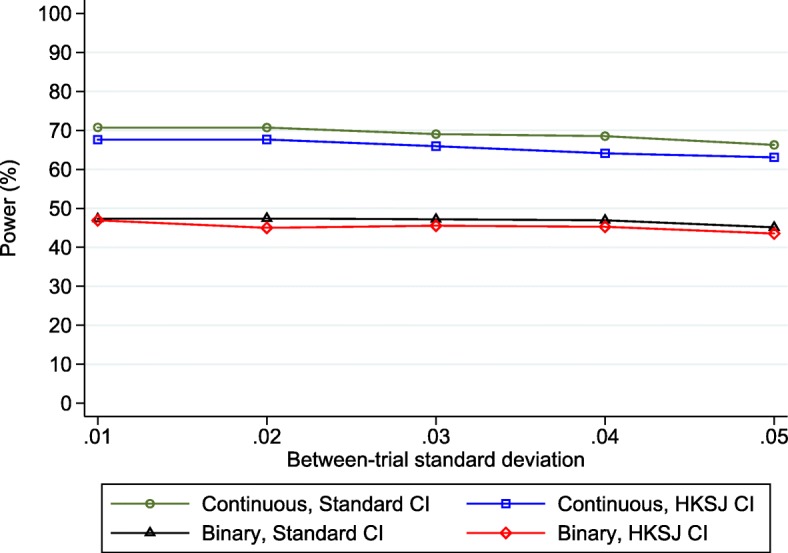
Simulation-based power estimates (based on 10,000 replications) for the planned IPD random effects meta-analysis* of 24 trials for detecting a treatment-BMI interaction when the true effect was − 0.1, conditional on a range of values of the between-study standard deviation (*τ*_*λ*_) of the interaction effect, when either correctly analysing BMI as continuous or when wrongly analysing BMI as binary (≥ 30 versus < 30). *Based on using change score model (4) in each trial followed by random effects meta-analysis model (9). Standard = DerSimonian and Laird estimation, with *p*-values and CIs derived using standard normal-based method; HKSJ = DerSimonian and Laird estimation, with p-values and CIs derived using approach of Hartung-Knapp Sidik-Jonkman

The findings are shown in Fig. 2, and the mean I^2^ value was between 10% and 13% for all scenarios. Immediately apparent is that the power gradually reduces as the size of the between-trial heterogeneity increases, and it is now about 70% or less across the range of *τ*_*λ*_values. This is alarming, as it signals a planned random effects IPD meta-analysis of the 24 trials would not have adequate power to detect an interaction of − 0.1, even with only low heterogeneity. For example, with *τ*_*λ*_ = 0.01 (mean I^2^ = 10%), the estimated power based on *p*-values and confidence intervals is 70.8% based on the standard normal-based approach, which is more than a 10% reduction in power compared to that for the fixed-effect meta-analysis given no heterogeneity (which was 83.2%, Fig. 1). Interestingly, this is mainly due to poor estimation of the between-study variance itself, as we observed an upward bias in the estimate of *τ*_*λ*_ across simulations leading to wider confidence intervals and thus reduced power than if *τ*_*λ*_was truly known. The bias is because *τ*_*λ*_is especially problematic to estimate well, as the corresponding I-squared is about 10% and the true *τ*_*λ*_of 0.01 is close to zero. This leads to large variation in estimates of *τ*_*λ*_across the 10,000 simulated datasets, and because variance estimates are bounded at zero, their average value has a notable upward bias. Consequently, we observe lower power when *τ*_*λ*_is estimated than if we truly knew *τ*_*λ*_. This reflects the impact of using a random-effects model.

The power is also consistently lower (by about 3%) when using the HKSJ approach rather than the standard approach (Fig. 2). This is expected, as standard 95% confidence intervals are typically too narrow (leading to a > 5% type I error rate), and the HKSJ correction aims to address this, usually leading to wider confidence intervals and larger *p*-values.

### Extension to consider BMI as a binary variable

Out of interest, we also considered the power of a two-stage IPD meta-analysis of all 24 trials that rather includes baseline BMI as a binary covariate. To do this, the IPD were again simulated according to model [13] and thus continuous BMI effects were set as the truth, and with a true interaction of − 0.1 assumed between the intervention and baseline BMI. However, upon application to the simulated IPD the two-stage IPD meta-analysis wrongly included baseline BMI as a binary covariate, with a BMI ≥ 30 classed as 1 and a BMI < 30 classed as 0. This dichotomisation corresponded to a true interaction of about − 0.65 kg between the intervention effect and binary BMI, such that the group of individuals with a BMI ≥ 30 have, on average, a 0.65 kg further reduction in weight gain by using the intervention rather than control, in comparison to those with a BMI < 30.

When there was no heterogeneity in the interaction effect, and a two-stage fixed effect IPD meta-analysis was applied to the simulated IPD from the 24 trials, the estimated power to detect this interaction was 60.5%. This is over a 20% reduction in power compared to when baseline BMI was analysed correctly as a continuous variable (83.2%), emphasising a huge loss of information by wrongly dichotomising BMI (Fig. 2). Indeed, the estimated power of 60.5% is now similar to that for the original IPD meta-analysis of just 14 trials when baseline BMI was analysed correctly as continuous (59.2%). Therefore, in this particular example, the loss of power by dichotomising baseline BMI in the IPD meta-analysis of 24 trials is similar to throwing away IPD from 10 trials. The cost of dichotomisation is well known in single studies [33, 34], and the results here emphasise that it also generalises to the IPD meta-analysis setting.

Findings are similar in the settings with heterogeneity in the interaction effect, with power estimates now less than 50% compared to about 65–70% when analysed correctly as continuous (Fig. 2).

### Consideration of an analysis of covariance approach

Due to the published information available in each trial, our power calculations assumed interaction estimates are derived from a change score analysis, as this was the typical approach taken and reported for each trial. These power estimates may be deemed conservative, as after IPD are obtained it is probable that interaction estimates could be derived from an ANCOVA, which is potentially more powerful. However, the correlation between final weight and baseline pregnancy weight is extremely high (often > 0.9) and Vickers and Altman note that: [35] “the efficiency gains of analysis of covariance compared with a change score are low when there is a high correlation (say r>0.8) between baseline and follow up measurements. This will often be the case, particularly in stable chronic conditions such as obesity. In these situations, analysis of change scores can be a reasonable alternative ...”. This reassures us that power calculations based on the change score approach are pertinent here. However, we would advocate that when IPD is obtained, the ANCOVA approach is the analysis of choice as it adjusts for any baseline imbalance in addition to improving power [19].

### Adjustment for additional covariates

Given the potentially inadequate power (< 70%, Fig. 2) when there is heterogeneity, it may be of interest to pre-specify the inclusion of additional covariates (prognostic factors) in the first stage of the two-stage IPD meta-analysis. Inclusion of prognostic factors would reduce the residual variance in each trial, leading to more precise interaction estimates and potentially larger power. So far the chosen size of residual variances (${\sigma}_i^2\Big)$ was based on the variance of weight gain, as reported in publications (Table 3); however, this is potentially conservative given that baseline BMI was also included as a covariate in the data generating model [13]. There are also other prognostic factors in this field, such as age and parity, which could be included.

**Fig. 3:**
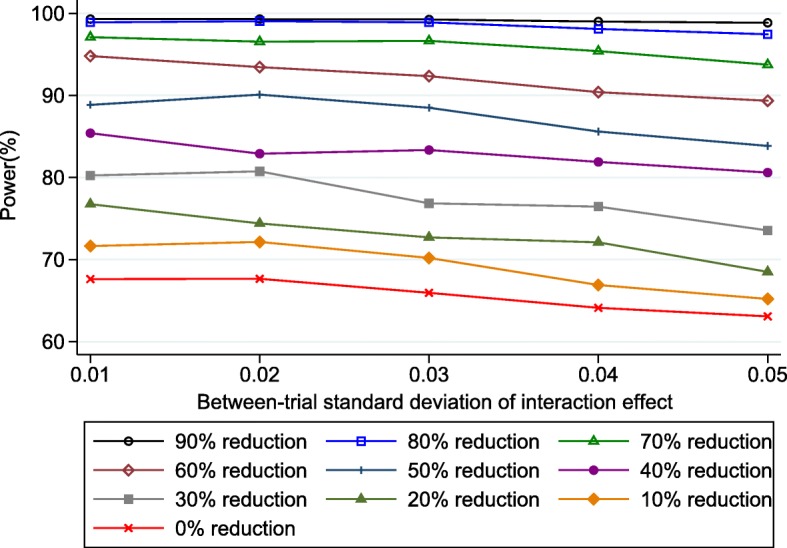
Simulation-based power estimates for the planned IPD random effects meta-analysis* of 24 trials for detecting a treatment-BMI interaction when the true effect was − 0.1, conditional on a range of values of the between-study standard deviation (*τ*_*λ*_) of the interaction effect, and a particular % reduction in residual variances in each trial due to the inclusion of prognostic factors. *Based on using change score model (4) in each trial followed by random effects meta-analysis model (9) with DerSimonian and Laird estimation, and *p*-values and CIs derived using approach of Hartung-Knapp Sidik-Jonkman

We therefore repeated our simulations of power in the IPD meta-analysis of 24 trials when residual variances were reduced by between 10% and 90% in each trial. For brevity, we again focus on a true interaction effect of − 0.1, across a range of values on the between-study standard deviation (*τ*_*λ*_). The results in Fig. 3 show that the power improves as the residual variances decrease, and thus pre-specified adjustment for prognostic factors is recommended. However, the power only consistently exceeds 80% across the entire range of *τ*_*λ*_ values when the reduction in residual variances is at least 40%.

Had this been known to the i-WIP researchers when planning their IPD project, it could have motivated them to identify the strongest prognostic factors in this field, and ascertain what the likely percentage reduction in residual variance by including them (e.g. by obtaining IPD from one trial and comparing the residual variances before and after inclusion of prognostic factors).

## Discussion

IPD meta-analyses are widely considered the gold standard in meta-analysis, and an increasing number are being funded to examine subgroup effects and interactions. However, it is currently rare to see power addressed in IPD meta-analysis grant applications or protocols. Yet power and sample size considerations are pivotal, as an IPD meta-analysis is costly and time-consuming, and so resources are better allocated to those projects that are adequately powered to detect effects of interest. Even when IPD are available for all studies, the power may not be adequate. Conversely, whilst ensuring selection biases are avoided [36], IPD may not be needed from all studies if a representative subset of trials has large power (e.g. > 95%), which could save considerable time, costs and frustration [7, 8].

To address this, here we outlined a simulation-based approach to power calculations for IPD meta-analysis that utilise a two-stage IPD meta-analysis framework. We demonstrated the approach for continuous outcomes, using a planned IPD meta-analysis of pregnancy trials (i-WIP), and showed that IPD from 14 trials was unlikely to have adequate power to detect a treatment-BMI interaction unless the effect was very large (Fig. 1). However, IPD from 24 trials was identified to have over 80% power to detect an interaction of at least − 0.1, assuming a fixed-effect meta-analysis was appropriate. Had this information been available at the time, it would have helped the i-WIP collaboration to justify the costs and resources needed to collate and meta-analyse IPD from 24 trials. Nevertheless, there would remain a concern that even low heterogeneity on the interaction effect would have reduced the power to 70% or less (Fig. 2) when a random-effects model was used. Therefore, we also showed the potential gain in power by including prognostic factors in the analysis, which would increase power to over 80% even with heterogeneity (Fig. 3), and thus motivates the identification and pre-specification of prognostic factors for inclusion in the IPD meta-analysis. If the true relationships for BMI are linear, the power calculations also made it clear that baseline BMI should be analysed as a continuous variable, as the power is reduced dramatically when BMI is wrongly (and arbitrarily) dichotomised at 30 (Fig. 3). Of course, *after* IPD is obtained, one may rather examine non-linear trends using splines for example. Our Stata code can be easily modified to generate IPD assuming non-linear trends and interactions, if that is considered plausible. However, unless there is evidence to the contrary, the assumption of linearity would appear a sensible starting point when considering potential power *prior* to the IPD being collected.

Our Stata module for the continuous outcome setting of the i-WIP meta-analysis is available in the supplementary material, and requires inputs as shown in Table 1 (see Additional file 1). Users will need to tailor this for their own IPD projects, as outlined by the four step process of Section 3. Extension to binary or survival outcomes would require consideration of event prevalence and event rates, respectively, and the latter would also require assumptions about the distribution of survival times (shape of hazard function), censoring and length of follow-up [37]. Table 4 provides key details about how to extend the approach to binary and time-to-event outcomes. Each IPD meta-analysis project is unique, and the simulation-based approach will need to be tailored to the information and setting at hand, as with standard power calculations for single trials. For example, in our application the mean and standard deviation of baseline BMI values were not known for all trials, and thus our module needed to generate BMI values differently for these trials compared to the others.

**Table 4:** Typical inputs required for simulation-based power calculations for an IPD meta-analysis of randomised trials with a binary or a time-to-event outcome, using a two-stage IPD framework

When considering the power of a summary (overall) treatment effect:	
• Number of IPD meta-analysis datasets to generate	
• Number of trials in the IPD meta-analysis	
• Number of patients in each trial, and proportion treated	
• Analysis model and method for estimating the treatment effect in each study separately	
• Distribution and magnitude of treatment effects across all trials, e.g. normal with a particular mean (summary) effect and between-trial variance	
• Approach to use in second stage of the two-stage IPD meta-analysis: e.g. fixed effect model (equation 5) or random effects model (equation 9)	
• Approach to derive confidence intervals and *p*-values (e.g. conventional method, Hartung-Knapp Sidik-Jonkman, etc)	
Binary outcomes	
• Baseline event risk in the control group in each trial (and any correlation between baseline risk and treatment effect across trials, if relevant)	
Time-to-event outcomes	
• Maximum length of follow-up in each trial	
• Distribution of event times in the control group in each trial, and whether these are related or change across trials (corresponding to the shape of the baseline hazard function in each trial and whether they are the same, different but proportional, or completely distinct)	
• Censoring mechanism and amount of censoring over time	
• Magnitude of any non-proportional hazards in treatment effect	
Additionally, when considering the power of a treatment-covariate interaction:	
• Analysis model and method for estimating the interaction effect in each study separately	
• Distribution and magnitude of covariate values in each trial; e.g. normal with chosen mean and variance for a continuous covariate, or Bernoulli for a binary covariate with a chosen probability of being a 1.	
• Between-trial distribution and magnitude of treatment-covariate interaction effect, e.g. normal with a particular (summary) mean effect and between-trial variance	
• Magnitude of any non-proportional hazards in interaction effect	

Simulation-based power calculations have been proposed by many others before us [38–40], including for random-effects models in general [41], and within the IPD meta-analysis field [12]. However, the novel aspect of our work is that it is based on a two-stage IPD meta-analysis framework [18, 42]. One-stage and two-stage approaches to IPD meta-analysis usually give similar results if their assumptions and estimation methods agree [18]. The main disadvantage of the two-stage approach is when there are rare events and/or small sample sizes, as then continuity corrections may be required and the assumption of ‘known’ within-study variances is likely to be inappropriate [18]. However, the two-stage approach also has many advantages. Firstly, it is relatively quick, and in particular facilitated by the excellent module ‘ipdmetan’ within Stata [43], which undertakes both stages automatically. Secondly, in the second stage it utilises well-known meta-analysis approaches, such as inverse variance weighted fixed effect and random effects analyses, and enables a variety of estimation methods, such as REML and the DerSimonian and Laird method as desired. Indeed, in our applied example we showed how the user can examine power for their own preferred approach and estimation methods. Thirdly, it allows novel options such as HKSJ for deriving *p*-values and confidence intervals, which have been shown to improve type I error rates (and thus will give more appropriate power results) [14, 15, 44]. Fourthly, and perhaps most importantly, it automatically avoids using across-trial information to inform treatment-covariate interactions, as these are estimated separately in each trial.

In contrast, one-stage models utilise both within-trial and across-trial information toward interaction estimates unless covariates are centred, and this would lead to wrongly inflated power estimates, as utilising across-trial information is strongly discouraged, being prone to ecological bias and study-level confounding [16, 17]. Indeed, two competing options to power calculations by Kontopantelis et al. [12] and by Kovalchik et al. [10, 11] utilise a one-stage IPD meta-analysis framework amalgamating within-trial and across-trial interactions. That being said, these are otherwise excellent alternative options for considering power for IPD meta-analysis, which use simulation or analytic methods. Our approach is somewhat faster than the ‘ipdpower’ module of Kontopantelis et al., as the two-stage framework is typically faster than the one-stage framework, due to the large number of parameters usually estimated simultaneously in the one-stage approach. Indeed, as noted by Kontopantelis et al. in their online help file, one-stage models are also prone to convergence problems, and for complex models (with multiple random effects) “non-convergence is more frequent than convergence.” The analytic approach of Kovalchik et al. is restricted to a fixed interaction effect, and so is limited when heterogeneity is of interest, and does not accommodate adjustment for prognostic factors. Further research comparing power in the context of two-stage and one-stage approaches would be welcome.

Simmonds and Higgins also provide algebraic solutions for the power of an IPD meta-analysis of continuous outcomes, under certain conditions, for both a one-stage IPD meta-analysis (that amalgamates within-trial and across-trial interactions) and a two-stage IPD meta-analysis [9]. However, these are based on strong assumptions, in particular no heterogeneity of overall treatment effects or interactions, the same number of patients in each treatment group within a trial, and same residual variances in all trials. The beauty of a simulation-based approach is that such assumptions can be easily relaxed, whereas an algebraic approach quickly becomes intractable, especially for non-normal outcomes. For example, simulations can be adapted to allow non-continuous outcomes (binary, survival, ordinal, etc), non-normal distributions for continuous covariates, multiple adjustment terms, non-linear trends, and multiple (even correlated) random-effects terms, as desired. This is at the expense of increased computational time, although 1000 simulations for our example would rarely take longer than 3 min for a particular set of inputs. The number of simulations required could be reduced in particular cases, with researchers able to calculate the number of simulations needed to achieve a given precision on the estimated power of their IPD meta-analysis. Our approach also could be extended to incorporate study-level covariates in the data generating model. This would allow true treatment and interaction effects in each trial to be tailored to study-level covariates, whereas we currently generate them randomly. Importantly, although we focussed on IPD meta-analysis of randomised trials, the simulation-based approach could be equally used to estimate power for other IPD meta-analysis research, such as prognostic factor research [45].

## Conclusions

In summary, we encourage researchers and funders to make assessments of power when planning or commissioning an IPD meta-analysis project. We propose a simulation-based approach to do this, utilising a two-stage IPD meta-analysis framework, as illustrated here for continuous outcomes. This overcomes the need for deriving analytic solutions, and is flexible enough to be tailored to each IPD meta-analysis project at hand. In particular, the user can evaluate power based on chosen statistical models and estimation methods, whilst utilising existing aggregate data about the set of trials promising their IPD. This informs how much IPD is required and helps reveal whether the IPD project is worth the investment.

## Additional file

Additional file 1: Stata simulation program code. Stata code to simulate power for IPD meta-analysis as proposed in this article. (PDF 158 kb)

## Abbreviations

ANCOVA: Analysis of covariance
BMI: Body mass index
HKSJ: Hartung-Knapp Sidik-Jonkmann
HTA: Health technology assessment
IPD: Individual participant data
i-WIP: Weight Management in Pregnancy International IPD Collaboration
ML: Maximum likelihood
MoM: Method of moments
NIHR: National Institute for Health Research
REML: Restricted maximum likelihood
