# Supplementary material for: Simulation-based power calculations for planning a two-stage individual participant data meta-analysis
Source: BMC Med Res Methodol. 2018 May 18;18:41. doi: 10.1186/s12874-018-0492-z (PMC5960205; doi:10.1186/s12874-018-0492-z)
Supplement: Supplementary file 1 — Stata simulation program code. Stata code to simulate power for IPD meta-analysis as proposed in this article. (PDF 158 kb) [file 12874_2018_492_MOESM1_ESM.pdf]

Stata code for generating simulation data used for this manuscript

```
* PART A: Data generating model

* define the program *
clear
capture program drop sim_pow
program define sim_pow, rclass

/* Syntax
            reps = Number of replications (simulations)
            s = Number of studies
            n = Number of subjects in each study
            e = Effect size
            treat_tausq = between-study variance of overall treatment
effect
            study_tausq = between-study variance of intercept (baseline
risk)
            alpha = constant term in the model
            interaction = interaction term in the model
            m = overall mean of the continuous factor for whole dist
            bsd = between study SD for continuous factor
            wsd = within study SD for continuous factor
            baseline_beta = prognostic effect of covariate
            inter_tausq = between-study variance of interaction effect
            type = fixed, random(dl), random(hk), random(reml)
*/
      syntax , reps(int) n(string) s(int) e(real) treat_tausq(real)
study_tausq(real) alpha(real) ///
                        interaction(real) baseline_beta(real) m(real)
bsd(real) wsd(real) ///
                        inter_tausq(real) res_var(real) type(string)

* display model being used *

di "Performing `type'-effects meta-analysis ..."

* Define number of repitions
global reps=`reps'

* Generate blank dataset to store raw results of simulation
clear
qui set obs $reps
qui gen trtEst=0
qui gen trtSe=0
qui gen trtPval=0
qui gen isq = 0
qui gen tausq = 0
qui gen converge=.
qui gen ncounter=_n
save res1, replace

* Start to loop over replications to simulate power
local k=1
while `k'<=$reps {

* Set observations to the number of subjects (per study) multiplied by the
number of studies
clear
local obs = `n'[1,1]
if `s'>1 {
```

```
      forvalues j = 2/`s' {
                  local obs = `obs' + (`n'[1,`j'])
            }
      }
qui set obs `obs'

* Generate ID and Study ID
qui gen id = _n
qui gen study = 1 if id<=`n'[1,1]
if `s'>1 {
      local counter = `n'[1,1]
            forvalues j = 2/`s' {
                        local counter = `counter' + (`n'[1,`j'])
                        qui replace study = `j' if study==. & id<=`counter'
                        }
            }

* Generating the baseline covariate (called base) for patients in each
study
qui gen base = .
forvalues j= 1/`s' {
* for trials where base mean and sd is not know, we can simulate from ///
* between and within study distributions based on other studies

            local studymean = rnormal(`m',`bsd')
            // btwn
            qui replace base = rnormal(`studymean',`wsd') if study==`j'
//within

* for some trials the mean and sd may be known, as in our example for BMI,
and
* so we replace the base values to be tailored ones

            qui replace base = rnormal( 34.75, sqrt(12.5) ) if `j' == 1 &
study == 1
            qui replace base = rnormal( 30.15, sqrt(25.51)) if `j' == 2 &
study == 2
            qui replace base = rnormal( 37.95, sqrt(0.49)) if `j' == 3 &
study == 3
            qui replace base = rnormal( 33.63, sqrt(14.77)) if `j' == 4 &
study == 4
            qui replace base = rnormal( 24.55, sqrt(27.45)) if `j' == 7 &
study == 7
            qui replace base = rnormal( 35.1, sqrt(12.25)) if `j' == 8 &
study == 8
            qui replace base = rnormal( 23.85, sqrt(0.25)) if `j' == 10 &
study == 10
            qui replace base = rnormal( 25.45, sqrt(13.45)) if `j' == 12 &
study == 12
            qui replace base = rnormal( 23.65, sqrt(18.13)) if `j' == 13 &
study == 13

                                    }
*identify the mean base value in each study
qui egen base_mean = mean(base), by(study)

* centre the base value in each trial
qui gen base_cent = base - base_mean

// Dichotomising the continuous patient-level covariate
// IN THIS EXAMPLE - BMI>=30 CLASSED AS OBESE PATIENTS
```

```
qui gen base_bin = 0
qui replace base_bin = 1 if base>=30

* Allocate patients in each study to treatment group
* here we have an equal probability of being in each group
* but could tailor to the exact numbers in each study
* in our example, it made little difference as groups were very even
* so here we include the most general form
qui gen trt = runiform()<.5
* generate an interaction with baseline, either between the binary base or
cts base
qui gen trt_base = trt*base_bin
qui gen trt_ctsbase = trt*base_cent

* Generating the mean outcome values using a linear model
* this is for the continuous covariate
* outcome 1 is the continuous outcome response

qui gen outcome1 =.
forvalues j=1/`s' {
* add random effect on the treatment effect
      local v = rnormal(0,sqrt(`treat_tausq'))
* add random effect on the study intercept
      local nu = rnormal(0,sqrt(`study_tausq'))
* add random effect on the interaction effect
      local interaction_i = rnormal(`interaction', sqrt(`inter_tausq'))

* draw outcome 1 from the following equation
      qui replace outcome1 = (`alpha'+`nu')+((`e'+`v')*trt) +
(`baseline_beta'*base_cent) ///
      + (`interaction_i'*trt_ctsbase)  if study==`j'

* here we replace outcome 1 for those trials where the control group mean
effect was known
      qui replace outcome1 = (13.3)+((`e'+`v')*trt) +
(`baseline_beta'*base_cent) ///
      + (`interaction_i'*trt_ctsbase)  if `j'==1 & study == 1

      qui replace outcome1 = (5)+((`e'+`v')*trt) +
(`baseline_beta'*base_cent) ///
      + (`interaction_i'*trt_ctsbase)  if `j'==2 & study == 2

      qui replace outcome1 = (9.68)+((`e'+`v')*trt) +
(`baseline_beta'*base_cent) ///
      + (`interaction_i'*trt_ctsbase)  if `j'==3 & study == 3

      qui replace outcome1 = (10.6)+((`e'+`v')*trt) +
(`baseline_beta'*base_cent) ///
      + (`interaction_i'*trt_ctsbase)  if `j'==4 & study == 4

      qui replace outcome1 = (11.5)+((`e'+`v')*trt) +
(`baseline_beta'*base_cent) ///
      + (`interaction_i'*trt_ctsbase)  if `j'==5 & study == 5

      qui replace outcome1 = (15.24)+((`e'+`v')*trt) +
(`baseline_beta'*base_cent) ///
      + (`interaction_i'*trt_ctsbase)  if `j'==6 & study == 6

      qui replace outcome1 = (14.2)+((`e'+`v')*trt) +
(`baseline_beta'*base_cent) ///
      + (`interaction_i'*trt_ctsbase)  if `j'==7 & study == 7
```

```
      qui replace outcome1 = (5.2)+((`e'+`v')*trt) +
(`baseline_beta'*base_cent) ///
      + (`interaction_i'*trt_ctsbase)  if `j'==8 & study == 8

      qui replace outcome1 = (5.00)+((`e'+`v')*trt) +
(`baseline_beta'*base_cent) ///
      + (`interaction_i'*trt_ctsbase)  if `j'==9 & study == 9

      qui replace outcome1 = (12.4)+((`e'+`v')*trt) +
(`baseline_beta'*base_cent) ///
      + (`interaction_i'*trt_ctsbase)  if `j'==10 & study == 10

      qui replace outcome1 = (13.8)+((`e'+`v')*trt) +
(`baseline_beta'*base_cent) ///
      + (`interaction_i'*trt_ctsbase)  if `j'==11 & study == 11

      qui replace outcome1 = (8)+((`e'+`v')*trt) +
(`baseline_beta'*base_cent) ///
      + (`interaction_i'*trt_ctsbase)  if `j'==12 & study == 12

      qui replace outcome1 = (15.70)+((`e'+`v')*trt) +
(`baseline_beta'*base_cent) ///
      + (`interaction_i'*trt_ctsbase)  if `j'==13 & study == 13

      qui replace outcome1 = (15.4)+((`e'+`v')*trt) +
(`baseline_beta'*base_cent) ///
      + (`interaction_i'*trt_ctsbase)  if `j'==14 & study == 14
      }

* now generate a residual for each patient, based on the residual variance
in that study
* firstly we could assume same residual variance for all trials
qui gen res = rnormal(0, sqrt(`res_var'))
* or we could tailor the residual variance for each trial, if it is known
qui replace res = rnormal(0, sqrt(43.25)) if study == 1
qui replace res = rnormal(0, sqrt(15.57)) if study == 2
qui replace res = rnormal(0, sqrt(119.26)) if study == 3
qui replace res = rnormal(0, sqrt(52.69)) if study == 4
qui replace res = rnormal(0, sqrt(16.98)) if study == 5
qui replace res = rnormal(0, sqrt(37.37)) if study == 6
qui replace res = rnormal(0, sqrt(33.89)) if study == 7
qui replace res = rnormal(0, sqrt(6.625)) if study == 8
qui replace res = rnormal(0, sqrt(12.93)) if study == 9
qui replace res = rnormal(0, sqrt(12.63)) if study == 10
qui replace res = rnormal(0, sqrt(15.22)) if study == 11
qui replace res = rnormal(0, sqrt(12.93)) if study == 12
qui replace res = rnormal(0, sqrt(13.78)) if study == 13
qui replace res = rnormal(0, sqrt(40.53)) if study == 14

* now we add the residual variance onto the outcome 1 value
qui gen outcome2 = outcome1 + res

* here we safe one set of IPD, as this may be useful for diagnostic
purposes after the moduel is finished
if $reps == 1 {
qui save IPDdata, replace
}
```

```
* PART B - perform a two-stage IPD meta-analysis via ipdmetan and safe the
results

// Two-stage approach with fixed effect meta-analysis model

if `"`type'"'=="fixed" {
      capture qui ipdmetan, study(study) keepall  pool(trt_ctsbase)
nograph: reg outcome2 trt c.base_cent trt_ctsbase

* save the interaction effect as beta_trt and related statistics
            local beta_trt = r(eff)
            local se_trt = r(se_eff)
            local z = r(eff)/r(se_eff)
            local trt_p = (1-normal(abs(r(eff)/r(se_eff))))*2
            local tau2 = r(tausq)

            qui use res1, replace
            qui replace trtEst= `beta_trt' if ncounter==`k'
            qui replace trtSe= `se_trt' if ncounter==`k'
            qui replace trtPval= `trt_p' if ncounter==`k'
            qui replace isq = 0 if ncounter==`k'
            qui replace tausq = `tau2' if ncounter==`k'

            qui save res1, replace

            di "`k' " _continue
            local k = `k'+1
            }


      if `"`type'"'== "random(dl)" {

      // Two-stage approach with random effects meta-analysis model
estimated using DL and standard CIs

      capture qui ipdmetan, re(dl) study(study) keepall interaction nograph
: reg outcome2 trt##c.base_cent


            local beta_trt = r(eff)
            local se_trt = r(se_eff)
            local z = r(eff)/r(se_eff)
            local trt_p = (1-normal(abs(r(eff)/r(se_eff))))*2
            local i2 = r(Isq)
            local tau2 = r(tausq)

            qui use res1, replace
            qui replace trtEst= `beta_trt' if ncounter==`k'
            qui replace trtSe= `se_trt' if ncounter==`k'
            qui replace trtPval= `trt_p' if ncounter==`k'
            qui replace isq = `i2' if ncounter==`k'
            qui replace tausq = `tau2' if ncounter==`k'

            qui save res1, replace

            di "`k' " _continue
            local k = `k'+1
            }
```

```
// Two-stage approach with random effects meta-analysis model estimated
using using REML and standard CI


if  `"`type'"'== "random(reml)" {
	capture qui ipdmetan, re(reml) study(study) keepall interaction
nograph  : reg outcome2 trt##c.base_cent


	local beta_trt = r(eff)
		local se_trt = r(se_eff)
		local z = r(eff)/r(se_eff)
		local trt_p = (1-normal(abs(r(eff)/r(se_eff))))*2
		local i2 = r(Isq)
		local tau2 = r(tausq)

		qui use res1, replace
		qui replace trtEst= `beta_trt' if ncounter==`k'
		qui replace trtSe= `se_trt' if ncounter==`k'
		qui replace trtPval= `trt_p' if ncounter==`k'
		qui replace isq = `i2' if ncounter==`k'
		qui replace tausq = `tau2' if ncounter==`k'

		qui save res1, replace

		di "`k' " _continue
		local k = `k'+1
		}



// Two-stage approach with random effects meta-analysis model estimated
using DL and HKSJ CI
// Stage 1 - Multi-level IPD model
if `"`type'"'== "random(hk)" {
	capture qui ipdmetan, re(hk) study(study) keepall interaction nograph
: reg outcome2 trt##c.base_cent


		local beta_trt = r(eff)
		local se_trt = r(se_eff)
		local z = r(eff)/r(se_eff)
		* r(k) is the number of studies
		local trt_p = (1-t(r(k)-1, abs(r(eff)/r(se_eff))))*2
		local i2 = r(Isq)
		local tau2 = r(tausq)

		qui use res1, replace
		qui replace trtEst= `beta_trt' if ncounter==`k'
		qui replace trtSe= `se_trt' if ncounter==`k'
		qui replace trtPval= `trt_p' if ncounter==`k'
		qui replace isq = `i2' if ncounter==`k'
		qui replace tausq = `tau2' if ncounter==`k'

		qui save res1, replace

		di "`k' " _continue
		local k = `k'+1
		}
```

```
}

qui use res1, replace

* based on stored results, count number of p-values significant
qui count if trtPval<0.05
local trtPcount=r(N)
local nos = _N
local trt_pcentPvals = string((`trtPcount'/`nos')*100)

* display the power *
di _n "The IPD meta-analysis has `trt_pcentPvals'% power to detect the true
interaction" _n
return scalar trtpow = `trt_pcentPvals'

* display useful summary information (e.g. mean estimates to ensure they
are unbiased etc)

qui su trtEst
return scalar mean_Est = r(mean)

qui su trtSe
return scalar mean_se = r(mean)

qui su isq
return scalar mean_isq = r(mean)

qui su tausq
return scalar mean_tausq = r(mean)

end
*********************************************** End of simulation program


/// Example of examining power across a range of scenarios
* the following provides results for Figure 1, power of fixed effect IPD
meta-analysis of 14 trials

clear
* define study sizes as a vector
mat sizes = (50, 931, 125, 85, 235, 327, 45, 12, 39, 142, 105, 84, 15, 124)
tempname pow
tempfile powerdata
postfile `pow' int_effect int_pow mean_Est mean_se mean_isq mean_tausq_int
using `powerdata', replace

* repeat power calculations across a range of true interaction effects from
-0.5 to -0.01
foreach j in -0.5 -0.4 -0.3 -0.2 -0.15 -0.1 -0.05 -0.025 -0.01 {

* specify the starting values for the simulation program, and run over
10000 replications
* some values, such as the study-specific intercepts and residual variances
for the 14 trials, are tailored during the sim code already
      sim_pow,  reps(10000) n(sizes) s(14) e(-0.84) treat_tausq(1.1)
study_tausq(22) alpha(11) ///
                        interaction(`j') baseline_beta(-0.28) m(30)
bsd(2.5) wsd(3.5) inter_tausq(0) ///
                        res_var(16) type(fixed)
```

```
      * post pertinent results to a dataset
      local int_effect=`j'
      local int_pow=`r(trtpow)'
      local mean_Est=`r(mean_Est)'

      local mean_se=`r(mean_se)'

      local mean_isq = `r(mean_isq)'
      local mean_tausq_int = `r(mean_tausq)'

      post `pow' (`int_effect') (`int_pow') (`mean_Est') (`mean_se')
(`mean_isq') (`mean_tausq_int')

      }


postclose `pow'
use `powerdata', replace

* plot the power across the different scenarios
twoway (line int_pow int_effect, sort lcol(red))  ,  xlabel(,
format(%9.2f))
```
